# Supplementary material for: Serum and CSF Biomarkers Predict Active Early Cognitive Decline Rather Than Established Cognitive Impairment at the Moment of RRMS Diagnosis
Source: Diagnostics (Basel). 2022 Oct 23;12(11):2571. doi: 10.3390/diagnostics12112571 (PMC9689215; doi:10.3390/diagnostics12112571)
Supplement: Supplementary file 1 [file diagnostics-12-02571-s001.zip › diagnostics-1844316-supplementary.pdf]

**Supplementary table S1 – Cognitive performance for each BDI II score category**			
**No. of patients**	**Minimal signs of depression (BDI II of 0 to 13 points)** (# of patients)	**Mild to moderate signs of depression (BDI II of 14 to 28 points)** (# of patients)	**Severe signs of depression (BDI II of 29 to 64 points)** (# of patients)
Baseline	43	7	0
One year follow-up	43	7	0
**Baseline testing**	**Mean values (min; max)**	**Mean values (min; max)**	**Statistical significance (p)**
MOCA	26.0 (17;30)	26.0 (20;30)	0.99
SDMT (oral)	45.3 (19;76)	51.1 (29;68)	0.63
BVMTR 1	5.4 (0;12)	6.0 (2;12)	0.71
BVMTR 2	8.1 (2;12)	8.7 (2;12)	0.70
BVMTR 3	9.5 (1;12)	9.9 (5;12)	0.74
BVMTR DR	9.3 (1;12)	9.7 (4;12)	0.74
**One year follow-up testing**	**Mean values (min; max)**	**Median values (min; max)**	**Statistical significance (p)**
MOCA	27.5 (17;30)	28.3 (25;30)	0.28
SDMT	55.5 (27;77)	52.9 (31;72)	0.63
BVMTR 1	6.6 (1;12)	7.3 (4;9)	0.41
BVMTR 2	9.3 (0;12)	10 (8;12)	0.34
BVMTR 3	10.5 (3;12)	10.7 (8;12)	0.75
BVMTR DR	10.1 (2;12)	10.9 (8;12)	0.30

Statistical significance is defined as a p value <0.05

Abbreviations: MoCA, Montreal Cognitive Assessment; SDMT, oral Symbol Digit Modalities Test; BVMTR, Brief Visuospatial Memory Test-Revised; BDI II, Beck Depression Inventory;

**Supplementary table S2 – Correlations between changes in BDI II score and cognitive performance**		
	**Changes in SDMT (oral)**	**Changes in MoCA**
Changes in BDI II score from baseline to one-year-follow-up	-0.25	0.04
P value	0.08	0.79

Statistical significance is defined as a p value <0.05

Abbreviations: MoCA, Montreal Cognitive Assessment; SDMT, oral Symbol Digit Modalities Test; BVMTR, Brief Visuospatial Memory Test-Revised; BDI II, Beck Depression Inventory;

Supplementary table S3 – Impact of education level on cognitive performance			
	**12 years of formal education**	**14-16 years of formal education**	
**Baseline testing**	Average (minimum; maximum)	Average (minimum; maximum)	p value
SDMT (oral)	44.4 (19;74)	47.1 (19;68)	0.50
MoCA	25.7 (19;30)	26.2 (17;30)	0.65
BVMTR 1	5.6 (0;12)	5.5 (0;12)	0.96
BVMTR 2	8.4 (3;12)	8.1 (2;12)	0.82
BVMTR 3	9.9 (7;12)	9.3 (1;12)	0.44
BVMTR DR	9.4 (3;12)	9.4 (1;12)	0.96
One year follow-up	Average (minimum; maximum)	Average (minimum; maximum)	p value
SDMT (Oral)	50.9 (31;76)	58.4 (27;74)	0.04
MoCA	26.9 (19;30)	28 (21;30)	0,18
BVMTR 1	7.0 (1;12)	6.2 (1;12)	0.41
BVMTR 2	9.7 (2;12)	9.0 (0;12)	0.46
BVMTR 3	10.5 (3;12)	10.6 (4;12)	0.88
BVMTR DR	10.2 (2;12)	10.3 (3;12)	0.86

Statistical significance is defined as a p value <0.05

Abbreviations: MoCA, Montreal Cognitive Assessment; SDMT, oral Symbol Digit Modalities Test; BVMTR, Brief Visuospatial Memory Test-Revised;

Supplementary material S4 - Cognitive decline in the first year following diagnosis – statistical analysis

An independentsamples t-test was run for continuous variables to determine if there were differences in the mean values of the biomarkers between the 2 groups (cognitive decline vs no cognitive decline). Selected variables (BREMSO, RoAD, all neurofilament samples, average RNFL and GCL+IPL layer thickness, CSF Aβ42) were normally distributed, with the exception of RoAD score (p=0,43) and CSF NfL (p=0,18) as assessed by the Shapiro-Wilk test.

There was homogeneity of variances, as assessed by Levene's test for equality of variances (p >0.05), with the exception of sNfL z-scores at baseline. There was a statistically significant difference in mean values between the 2 groups for sNfL z-scores at baseline (a Welch t-test was run for this variable due to unequal variances), t(43)=3.32, p=0,006, as well as for sNfL z-scores at 3 months follow-up t(47)=2.34, p=0.02.

A Mann-Whitney U test was run to determine if there were differences in CSF NfL at baseline between the 2 cognition groups. Distributions were not similar, as assessed by visual inspection. CSF NfL values were higher for the cognitive decline group (mean rank = 35.67) versus the no decline group (mean rank = 21.05), U = 193, z = 2.538, p = .009, using an exact sampling distribution for U (Dineen & Blakesley, 1973).

An exact Fisher's test was run to test for differences in proportions between the 2 cognitive groups and the following dichotomous variables: all MRI metrics, presence of OCBs, positive immunoglobulin G index and education status. None showed statistical significance.

Due to previous reports on the cumulative predictive power of multiple NfL samples, we decided to test whether the pooled values of sNfL z-scores at baseline and 36 months follow-up would show a statistically significant difference between the 2 cognitive groups. We than ran a Hotelling's T2 (one-way MANOVA) for cognitive decline in the first year using sNFL z-scores at baseline, 3 and 6 months as dependent variables. We decided not to use other covariates, as preliminary analysis showed no correlation between mood disorders or education level and cognitive decline in the first year of follow-up, and age was already included as a factor in computing the sNfL z-scores.

There was no evidence of multicollinearity, as assessed by Pearson correlation ($|r| < 0.9$). Following boxplot analysis, we decided to remove 5 univariate outliers for the sNfL z-score at baseline, and 2 univariate outliers for the 3 months follow-up sNfL z-scores. After these adjustments, there were no multivariate outliers in the data, as assessed by Mahalanobis distance ($p > .001$).

Data was normally distributed for both groups (cognitive decline vs. no cognitive decline), as assessed by Shapiro-Wilk's test ($p > .05$). There was homogeneity of variances, as assessed by Levene's Test of Homogeneity of Variance ($p > .05$).

Patients with cognitive decline showed higher z-scores for all sNfL samples collected (2.7 ± 0.39 on baseline, 2.0 ± 0.42 at 3 months follow-up and 1.3 ± 0.5 at 6 months follow-up) versus patients with no cognitive decline (1.8 ± 0.15 on baseline, 1.4 ± 0.16 at 3 months follow-up, and 1.0 ± 0.2 at 6 months follow-up).

The differences between the 2 groups on the combined dependent variables was not statistically significant, $F(3, 41) = 1.745$, $p = 0.173$; Wilks' $\Lambda = 0.887$; partial $\eta 2 = 0.113$.
